# Sensitivity to Pain Traumatization and Its Relationship to the Anxiety–Pain Connection in Youth with Chronic Pain: Implications for Treatment

**DOI:** 10.3390/children9040529

**Published:** 2022-04-08

**Authors:** Larah Maunder, Maria Pavlova, Jaimie K. Beveridge, Joel Katz, Tim V. Salomons, Melanie Noel

**Affiliations:** 1 Department of Psychology, Queen’s University, Kingston, ON K7L 3N6, Canada; tim.salomons@queensu.ca; 2 Department of Psychology, University of Calgary, Calgary, AB T2N 1N4, Canada; mpavlova@ucalgary.ca (M.P.); jaimie.beveridge@ucalgary.ca (J.K.B.); melanie.noel@ucalgary.ca (M.N.); 3 Department of Psychology, York University, Toronto, ON M3J 1P3, Canada; jkatz@yorku.ca; 4 Alberta Children’s Hospital Research Institute, Calgary, AB T2N 4N1, Canada; 5 Hotchkiss Brain Institute, Calgary, AB T2N 4N1, Canada; 6 Mathison Centre for Mental Health Research and Education, Calgary, AB T2N 4Z6, Canada

**Keywords:** sensitivity to pain traumatization, pediatric chronic pain, anxiety, moderation analysis

## Abstract

The bidirectional relationship between anxiety and chronic pain in youth is well-known, but how anxiety contributes to the maintenance of pediatric chronic pain needs to be elucidated. Sensitivity to pain traumatization (SPT), an individual’s propensity to develop responses to pain that resemble a traumatic stress response, may contribute to the mutual maintenance of anxiety and pediatric chronic pain. A clinical sample of youth (aged 10–18 years) with chronic pain completed a measure of SPT at baseline and rated their anxiety and pain characteristics for seven consecutive days at baseline and at three-month follow-up. Multiple linear regression analyses were conducted to model whether SPT moderated the relationship between baseline anxiety and pain intensity, unpleasantness, and interference three months later. SPT significantly moderated the relationship between anxiety and pain intensity. High anxiety youth with high SPT reported increased pain intensity three months later, while high anxiety youth with low SPT did not. High anxiety youth who experience pain as potentially traumatizing are more likely to report higher pain intensity three months later than high-anxiety youth who do not. Future research should examine whether children’s propensity to become traumatized by their pain predicts the development of chronic pain and response to intervention.

## 1. Introduction

Chronic pain is highly prevalent in children and adolescents, with prevalence rates ranging from 11 to 38% [1]. Pediatric chronic pain is associated with high rates of school absenteeism [2], limited functioning in everyday activities [3], and worse quality of life [4]. The condition is not limited to childhood; it can persist [5] and lead to the development of mental health disorders well into adulthood [6]. Therefore, identification of risk factors for pediatric chronic pain is key, as they may provide early intervention targets to prevent pain from persisting into adulthood.

Anxiety is one psychological factor that contributes to and exacerbates chronic pain [7,8]. Chronic pain and anxiety symptoms co-occur at high rates in youth and adults [6,9,10,11,12,13,14], and trait anxiety is also implicated in chronic pain. For instance, trait-level worry and rumination have been associated with pain intensity [15]. Thus, the relationship between anxiety and pediatric chronic pain is well-established; however, the mechanisms through which anxiety facilitates the development and maintenance of chronic pain still need to be identified. Sensitivity to pain traumatization (SPT) may be one such psychological mechanism through which anxiety contributes to and exacerbates chronic pain. SPT is the propensity to develop anxiety-related somatic, cognitive, emotional, and behavioral responses to pain that resemble features of a traumatic stress response [16,17]; it is a general, higher-order factor underlying the pain-related anxiety constructs of anxiety sensitivity, pain anxiety, and pain catastrophizing [16,17]. SPT is similar to posttraumatic stress disorder (PTSD) symptomatology in that it involves a traumatic stress reaction, yet is distinct from it in that pain itself is conceptualized as a traumatic stressor, regardless of whether or not an individual has experienced a painful and/or traumatic event [16]. Its relation to PTSD differentiates SPT from other pain-related anxiety constructs, as several SPT symptoms correspond to PTSD symptoms and clusters (e.g., experiencing/intrusive thoughts, avoidance, hyperarousal, pain dissociation) [17]. SPT has been proposed as one psychological construct that may magnify chronic pain in youth and adults [16,18], because having a high susceptibility to developing a traumatic stress-like reaction if/when pain is experienced may directly exacerbate chronic pain. Youth who are exposed to chronic pain, a painful injury, or surgery may develop frequent and intense traumatic stress-like symptoms (e.g., hyperarousal, terror in response to pain sensations, intrusive pain memories). These symptoms might directly contribute to pain and disability.

In addition, anxiety-related constructs (such as neuroticism and pre-trauma arousal) have been identified as risk factors for the development of posttraumatic stress symptoms in adolescents and adults [19,20]. Given that SPT involves traumatic stress-like symptoms, youth with chronic pain who are high in anxiety relative to those low in anxiety might be more susceptible to experiencing pain traumatization when they experience pain, and in turn, more severe chronic pain. This relationship may also be bidirectional, in that high SPT may potentiate anxiety processes and symptoms (e.g., increased bodily tension, worry) that exacerbate chronic pain or contribute to its development and maintenance. Thus, youth with chronic pain who are high in anxiety may be more susceptible to developing pain traumatization, and vice versa, which may magnify their chronic pain.

Only one other study has examined SPT in youth and demonstrated that higher baseline SPT scores predicted higher pain interference three months later [18]. However, the study did not investigate whether SPT acts as a moderator of the pediatric anxiety–chronic pain relationship. Thus, the current prospective study investigated whether baseline SPT moderated the relationship between baseline anxiety and pain outcomes at three-month follow-up in a clinical sample of youth with chronic pain. Given previous research that has found that different pain outcomes were associated with different long-term outcomes for youth (e.g., chronic pain status, severity of postsurgical pain; [21]), we investigated three pain outcomes in our models (i.e., pain intensity, unpleasantness, and interference). It was hypothesized that baseline SPT would predict pain and moderate the relationship between baseline anxiety and pain three months later. Such a result would suggest that youth high in anxiety compared to youth low in anxiety tend to have a higher susceptibility to experiencing pain traumatization and worse chronic pain.

## 2. Materials and Methods

The current study’s data was part of a longitudinal research program investigating mechanisms of co-occurring pediatric chronic pain and mental health symptoms. The aims of the current study were distinct from previously published articles that have used data from this study, e.g., [22,23,24,25,26].

### 2.1. Participants

In total, 184 youth aged 10–18 years (46 boys, 130 girls, 8 nondisclosed) from the outpatient multidisciplinary chronic pain programs at Alberta Children’s Hospital (Calgary, Alberta) were recruited for this study. A total of 40 participants either did not complete the research study or had incomplete questionnaire data at time 2, leading to a sample size of 144 to 151 at time 2 (depending on the specific statistical analysis conducted). One of each participant’s parents was also enrolled in the study and provided sociodemographic information about their child. Youth participants had a variety of chronic pain conditions, such as headache, abdominal, and musculoskeletal pain. Because youth were recruited from multidisciplinary chronic pain programs, many participants received chronic pain intervention during their participation in the research study. Given the unique symptoms, pain locations, and needs of each participant, intervention was unique to each participant.

#### 2.1.1. Inclusion Criteria

Youth participants were included in the study if they: (1) were identified by a provider in the pain clinics as having chronic pain (pain for at least three months) without a clear pathological explanation (e.g., participants with juvenile arthritis were considered to have chronic pain with a clear pathological explanation and were excluded from the study); and (2) reported ongoing pain upon recruitment screening.

#### 2.1.2. Exclusion Criteria

Youth were ineligible for the study if they: (1) were unable to read and speak in English; (2) had severe cognitive impairment, a developmental disorder, schizophrenia spectrum or other psychotic disorder, and/or a serious chronic health or life-threatening condition (e.g., cancer); and (3) were unable to access the internet.

### 2.2. Procedure

All study procedures were approved by the University of Calgary Conjoint Health Research Ethics Board (REB #15-3100). Research staff contacted potential participants who had previously indicated interest in participating in research studies via phone from the outpatient programs at Alberta Children’s Hospital. During the phone call, staff provided information about the study, invited potential participants to enroll, and screened them for eligibility. Eligible participants provided verbal consent to participate in the study. Electronic consent forms were subsequently sent via email to consenting families to obtain written informed consent. Youth participants below the age of 14 years provided their assent to participate, while their parents provided written informed consent for their child to participate. Youth participants aged 14 years or older provided their own consent to participate.

The study consisted of two timepoints. At baseline (time 1), youth participants completed the Sensitivity to Pain Traumatization Scale—Child Version (SPTS-C). Parents completed a brief measure of their child’s sociodemographic information (i.e., child’s age, gender, and race/ethnicity). For seven consecutive evenings at baseline, youth participants completed an electronic survey (entitled the Youth Daily Survey), developed for the current study from a series of validated rating scales, to assess how much anxiety, pain intensity, pain unpleasantness, and pain interference the participant had experienced that day. Links to the surveys were sent via email or text message (based on the participant’s preference) at 18:00 each evening, with instructions to complete the survey before bed. At time 2, which occurred three months after baseline assessment, youth participants once again completed seven consecutive days of the Youth Daily Survey to rate their daily pain intensity, pain unpleasantness, pain interference, and anxiety. All measures were completed via Research Electronic Data Capture (REDCap), a web-based data collection tool that uses a secure web connection, authentication, and data logging [27]. Youth participants received a CAD 20 gift card for their participation at each time point.

### 2.3. Measures

#### 2.3.1. Sensitivity to Pain Traumatization Scale—Child Version (SPTS-C)

The Sensitivity to Pain Traumatization Scale-12 (SPTS-12) [17] assesses the propensity of the respondent to develop anxiety-related somatic, cognitive, emotional, and behavioral responses to pain that are similar to a traumatic stress response. It consists of 12 items that are rated on a scale from 0 (“Not at all true”) to 4 (“Entirely true”). Scores on the questionnaire range from 0 to 48, with higher scores indicating greater sensitivity to pain traumatization. The child version of the SPTS-12, the SPTS-C, contains all SPTS-12 items, reworded to be age-appropriate and easily understood by children aged 8–18 years [18] (e.g., “When I’m in pain, things don’t feel real,” and “I try not to do activities that make the pain start.”). The scale has demonstrated good convergent validity, discriminant validity, and criterion validity [18]. This sample demonstrated good internal consistency, Cronbach’s α = 0.88.

#### 2.3.2. Youth Daily Survey

Anxiety. Youth participants rated their daily anxiety by responding to the prompt: “How anxious/nervous did you feel today?” Responses were rated on an 11-point numeric rating scale ranging from 0 (“Not at all anxious/nervous”) to 10 (“Extremely anxious/nervous”). The validity of 11-point numeric rating scales to measure anxiety in youth has been demonstrated in previous research [28].

Pain Intensity. Pain intensity (i.e., “How much pain did you have today?”) was rated on an 11-point numeric rating scale ranging from 0 (“No pain”) to 10 (“Worst pain possible”). The reliability and validity of this 11-point scale for measuring pain intensity in youth has been demonstrated in multiple studies [29,30].

Pain Unpleasantness. Pain unpleasantness (i.e., “How much has your pain bothered you today?”) was rated on a 5-point Likert-type scale ranging from 0 (“Not at all”) to 4 (“Very much”). This measure has adequate validity in youth, and has been used in previous research [31,32].

Pain Interference. Pain interference was assessed using the 4-item version of the Pain Interference Scale from the Patient-Reported Outcomes Measurement Information System (PROMIS-25) Pediatric Profile (version 1.0) [33]. Pain interference comprised the sum of 4 items that assessed the extent to which youth participants’ pain made it difficult for them to sleep, pay attention, run, and walk one block during the day. Each of these 4 facets of interference were rated on a 5-point numeric rating scale from 0 (“Never”) to 4 (“Almost Always”), resulting in a minimum score of 0, and a maximum score of 16. A similar short form of the Pain Interference Scale demonstrated validity in youth with chronic pain [34].

### 2.4. Data Preparation

For the anxiety measure, ratings over the 7-day study period at baseline were averaged to determine mean anxiety at baseline. For each pain measure, one average across the 7-day study period was computed at each time point (baseline and time 2). In other words, pain intensity ratings over the 7-day study period at baseline were averaged to determine mean pain intensity at baseline. Pain intensity ratings over the 7-day study period at time 2 were averaged to determine mean pain intensity at time 2. The same computations were also completed for pain unpleasantness and pain interference.

A total SPT score was not calculated for participants who had missing items on the SPTS-C, which was completed at baseline (i.e., these participants were excluded from the analyses). For each variable that was rated daily on the Youth Daily Survey (i.e., anxiety, pain intensity, pain unpleasantness, and pain interference), which was completed at baseline and again at time 2, participants must have provided at least one rating (out of 7) for that variable at baseline, and at least one rating (out of 7) for that variable at time 2, for their data to be included in the regression model. Participants with a missing value for a variable were not included in regression analyses that used that variable (i.e., listwise deletion of cases was carried out for missing values).

### 2.5. Statistical Analyses

Three multiple linear regression analyses were conducted using RStudio Version 1.1.383 to investigate whether SPT (SPTS-C) moderated the relationship between anxiety at baseline (Youth Daily Survey) and pain intensity, unpleasantness, and interference (Youth Daily Survey) at time 2 in youth. Given our interest in temporal relations between SPT and the anxiety–pain relationship, we were also interested in whether SPT moderated the pain–anxiety relationship on a day-to-day basis. Thus, we investigated whether SPT scores moderated the daily anxiety–daily pain relationship at baseline and the daily anxiety–daily pain relationship at time 2 using multilevel modeling. The multilevel model methods and detailed results can be found in the Supplemental Material.

#### 2.5.1. Model 1: Pain Intensity

We first investigated whether baseline anxiety was related to pain intensity three months later, and whether this relationship was moderated by self-reported SPT. To do this, we conducted a multiple linear regression employing baseline anxiety and baseline SPT score as predictor variables, and pain intensity at time 2 as the criterion variable. Baseline SPT score was also used as the moderating variable. To control for the effect of baseline pain intensity on time 2 pain intensity, baseline pain intensity was employed as an additional predictor variable.

#### 2.5.2. Model 2: Pain Unpleasantness

For Model 2, we conducted a multiple linear regression employing baseline anxiety, baseline SPT score, and baseline pain unpleasantness as predictor variables, pain unpleasantness at time 2 as the criterion variable, and baseline SPT score as the moderating variable.

#### 2.5.3. Model 3: Pain Interference

Model 3 comprised a multiple linear regression employing baseline anxiety, baseline SPT score, and baseline pain interference as predictor variables, pain interference at time 2 as the criterion variable, and baseline SPT score as the moderating variable.

For all models, interaction terms were created by multiplying baseline SPT score with baseline anxiety; this term served as the moderating variable in each model. Significant interaction effects were followed-up using a simple slopes analysis, which modeled the simple anxiety–pain slope at high levels of SPT (i.e., 1 standard deviation above mean SPT score) and low levels of SPT (i.e., 1 standard deviation below mean SPT score) [35]. This procedure allowed us to investigate the effect of SPT on the relationship between baseline anxiety and pain at time 2 at high and low levels of SPT. In addition, Johnson–Neyman analyses were applied to models with a significant interaction effect, to determine the level of SPT at which the relationship between anxiety and pain became significant [36].

## 3. Results

### 3.1. Descriptive Statistics

In the current sample, 71% were girls, and the average age was 14.21 years (*SD* = 2.23, range = 10–18). Average pain duration was 2.69 years (*SD* = 3.04, range = 0.25–12). Headache was the most reported chronic pain (72%), followed by other pain (25%) and muscle and joint pain (24%). Demographic and pain characteristics of the sample are summarized in Table 1. Pain outcome and anxiety descriptive statistics of the sample are summarized in Table 2. Of note, independent sample *t*-tests revealed that there were no significant differences between participants who completed the research study and attrition participants on study variables and demographic characteristics of interest (i.e., mean pain characteristics, pain duration, age, gender), except for pain unpleasantness at time 2. Participants who completed the study had significantly lower pain unpleasantness scores at time 2 compared to attrition participants who had pain unpleasantness scores for this timepoint (mean difference = −0.74).

### 3.2. Tests of Multivariate Assumptions

All regression models were assessed for fit between the model, its predictors, and the assumptions of multiple linear regression. This process allowed us to determine each model’s applicability to the population of youth with chronic pain. Model 1 (Pain Intensity) met all assumptions of multiple linear regression. For Model 2 (Pain Unpleasantness), the histogram and Q-Q plot of standardized residuals suggested a positively skewed distribution of errors. Confirming a departure from normality, the Shapiro–Wilk test was significant, *p* = 0.01. Pain unpleasantness at time 2 was therefore log-transformed. The score test for non-constant error variance was significant, *p* = 0.049, suggesting that there was heterogeneity in the variance of residuals. Therefore, the standard errors of the regression coefficients were calculated using a heteroscedasticity-corrected covariance matrix. All other assumptions of multiple linear regression for Model 2 were met. For Model 3 (Pain Interference), the histogram and Q-Q plot of standardized residuals suggested a positively skewed distribution of errors. Confirming a departure from normality, the Shapiro–Wilk test was significant, *p* = 0.02. Pain interference at time 2 was therefore log-transformed. The scatterplot of residuals (using log-transformed time 2 pain interference data) and the addition of a quadratic effect for mean anxiety indicated that there was not a linear relationship between anxiety and pain interference at time 2. To correct for this nonlinear relationship, anxiety was log transformed. All other assumptions of multiple linear regression for Model 3 were met.

### 3.3. Multiple Linear Regression Results

Correlations among all variables are presented in Table 3. The results of the three regression models are presented in Table 4.

#### 3.3.1. Model 1: Pain Intensity

**Figure 1:**
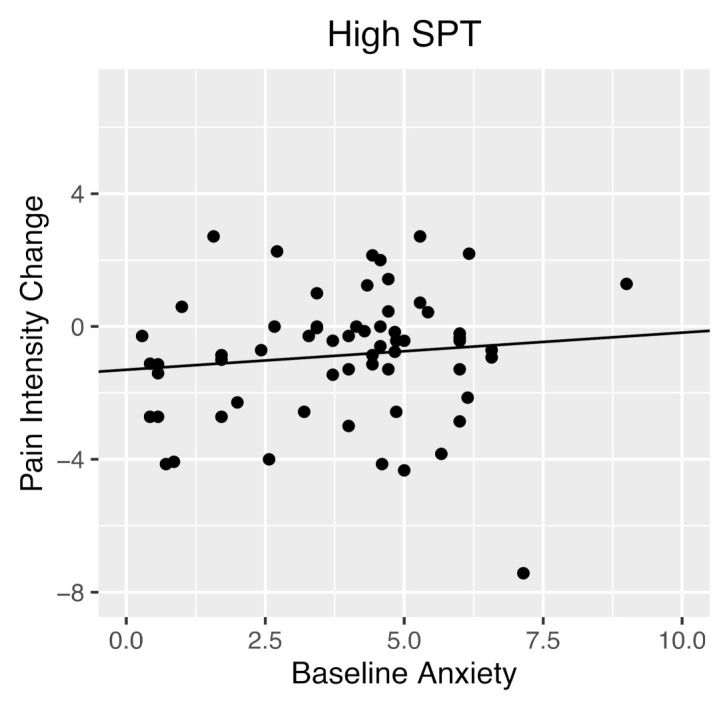
Simple slope of the relationship between baseline anxiety and pain intensity at three-month follow-up for youth with above average SPT (High SPT). Youth who were high in anxiety and had high SPT scores at baseline had higher pain intensity three months later as compared to youth who were high in anxiety but had low SPT scores. For graphing purposes, change scores are used to depict pain intensity at three-month follow-up, to graphically control for baseline pain intensity. SPT, sensitivity to pain traumatization.

**Figure 2:**
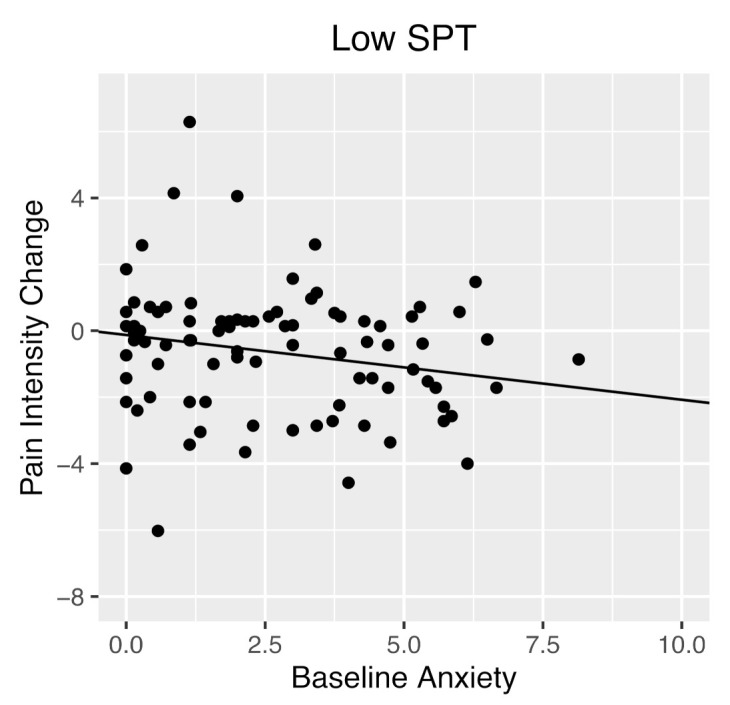
Simple slope of the relationship between baseline anxiety and pain intensity at three-month follow-up for youth with below average SPT (Low SPT). Youth who were high in anxiety and had low SPT scores at baseline had lower pain intensity three months later as compared to youth who were high in anxiety but had high SPT scores. For graphing purposes, change scores are used to depict pain intensity at three-month follow-up, to graphically control for baseline pain intensity. SPT, sensitivity to pain traumatization.

The multiple linear regression with baseline pain intensity, baseline anxiety, baseline SPT, and the baseline anxiety x SPT interaction effect predicting pain intensity at time 2 was significant, *R*^2^ = 0.50, *adjusted R*^2^ = 0.48, *F*(4, 146) = 35.74, *p* < 0.001. Baseline pain intensity significantly positively predicted pain intensity three months later, *b* = 0.69, *t*(146) = 9.87, *p* < 0.001. The main effects of baseline anxiety, *p* = 0.25, and baseline SPT, *p* = 0.14, were not significantly different from zero. As hypothesized, baseline SPT significantly moderated the relationship between baseline anxiety and pain intensity 3 months later, *b* = 0.02, *t*(146) = 2.02, *p* = 0.04. Simple effects showed that as baseline anxiety increased, those high on SPT reported an increase in pain intensity. Conversely, those low on SPT reported decreased pain intensity as baseline anxiety increased (Figure 1 and Figure 2). Johnson–Neyman analyses indicated that the relationship between baseline anxiety and time 2 pain intensity became significant when SPT score was 21.45 (out of 48) or higher.

#### 3.3.2. Model 2: Pain Unpleasantness

The multiple linear regression predicting pain unpleasantness at time 2 from baseline pain unpleasantness, baseline anxiety, baseline SPT, and the baseline anxiety x SPT interaction was significant, *R*^2^ = 0.42, *adjusted R*^2^ = 0.41, *F*(4, 141) = 26.07, *p* < 0.001. Baseline pain unpleasantness significantly predicted pain unpleasantness at time 2, *b* = 0.10, *t*(141) = 6.48, *p* < 0.001, but baseline anxiety and SPT were not significantly associated with pain unpleasantness at time 2, *p* > 0.20 for both predictors. The baseline anxiety x SPT interaction was also not significantly different from zero, *p* = 0.12, indicating that SPT did not moderate the relationship between baseline anxiety and pain unpleasantness at time 2.

#### 3.3.3. Model 3: Pain Interference

The multiple linear regression predicting pain interference at time 2 from baseline pain interference, baseline anxiety, baseline SPT, and the baseline anxiety x SPT interaction was significant, *R*^2^ = 0.48, *adjusted* *R*^2^ = 0.47, *F*(4, 139) = 32.2, *p* < 0.001. Baseline pain interference significantly predicted pain interference at time 2, *b* = 0.058, *t*(139) = 8.74, *p* < 0.001. Baseline anxiety was not significantly associated with pain interference at time 2, *p* > 0.20, nor was SPT, *b* = −0.018, *t*(139) = −1.97, *p* = 0.051. Baseline SPT did not significantly moderate the relationship between baseline anxiety and pain interference at time 2, *b* = 0.025, *t*(139) = 1.97, *p* = 0.051.

### 3.4. Multilevel Model Results

Results of the multilevel models that investigated whether SPT moderated the daily anxiety–daily pain relationship at baseline and at time 2 showed no significant cross-level interaction between SPT and daily anxiety at baseline or at time 2. This means that SPT did not significantly predict variation in the daily anxiety–daily pain slope at either time-point. However, SPT was significantly positively related to mean pain intensity, unpleasantness, and interference at baseline. At time 2, baseline SPT was significantly positively related to mean pain unpleasantness and interference, but not mean pain intensity. Detailed results can be found in the Supplemental Material.

## 4. Discussion

The current study investigated whether baseline SPT moderated the relationship between anxiety at baseline and pain intensity, unpleasantness, and interference three months later, while holding baseline pain intensity, unpleasantness, and interference constant (respectively), in a clinical sample of youth with chronic pain. We found partial support for our hypothesis, in that SPT significantly moderated the relationship between anxiety at baseline and pain intensity three months later. Youth who were high in anxiety and more sensitive to developing pain traumatization at baseline had higher pain intensity three months later as compared to youth who were high in anxiety but less sensitive to developing pain traumatization.

Given that neither the main effect of SPT nor that of anxiety predicted pain levels three months later, this suggests that evaluating the SPT x anxiety relationship in children with chronic pain is important, as knowledge of SPT severity among highly anxious youth may help predict which youth are likely to experience a worsening of pain severity over time. Identification of such youth would allow clinicians to apply early intervention strategies aimed at interrupting the associations among anxiety, pain traumatization, and pain, thus preventing pain from becoming worse over time. For instance, therapeutic treatments that aim to reduce youths’ pain traumatization symptoms, such as intrusive pain memories, hyperarousal, and dissociative experiences resulting from pain, may decrease the amount of distress that youth experience from their pain, ameliorate their current pain severity, and decrease the severity of their future pain flare-ups.

How might SPT mechanistically contribute to chronic pain or exacerbate the relationship between anxiety and chronic pain in youth? SPT is similar to PTSD in that it involves a response to pain that is similar to a traumatic stress reaction (e.g., avoidance of stimuli associated with the trauma, sleep difficulties). It is possible that the physiological changes that occur during this reaction (e.g., hypervigilance to pain) may transmit potential injury signals to the brain, which may then trigger pain perception as a mechanism to protect the individual [37].

It is also possible that SPT directly contributes to chronic pain in much the same way that PTSD is theorized to develop and maintain itself. According to the emotional processing theory of PTSD, fear and avoidance of trauma reminders serve to develop and maintain posttraumatic stress symptoms (PTSS) [38]. This is because avoidance of objectively safe trauma reminders due to fear prevents individuals from adequately emotionally processing the trauma reminders and experiencing symptom dissipation [38]. In the same way, a child who is more sensitive to becoming traumatized by their pain, or who has already developed a traumatic stress-like reaction to their pain, may be especially prone to avoiding pain “trauma reminders” (e.g., a particular body movement that triggers their chronic pain symptoms or an especially salient pain episode, or environments in which pain has been experienced). This may directly contribute to and exacerbate their chronic pain long-term by preventing the child from confronting and emotionally processing the pain trauma reminder, and in turn, learning that trauma reminders are objectively safe (i.e., do not cause bodily injury). Without this learning, traumatic stress-like SPT symptoms will be maintained, in turn exacerbating chronic pain. For instance, intrusive thoughts of pain may activate the sympathetic nervous system, in turn worsening chronic pain symptoms. In addition, avoidance of pain trauma reminders lowers the threshold at which affected youth feel pain when they eventually face a pain trauma reminder and decreases musculoskeletal strength and/or flexibility [39]. High SPT may also potentiate other anxiety processes that facilitate the development and maintenance of chronic pain, such as somatosensory amplification, increased bodily tension, and increased fear of movement [11]. This suggests that it may be possible to reduce chronic pain symptoms in children by not only gradually exposing youth to their feared pain situations (i.e., their pain trauma reminders) to encourage learning that the trauma reminder is objectively safe, but by also providing an environment in which they can process the emotions that arise immediately after the exposure. This will encourage emotional processing of the pain trauma reminder. Simons and colleagues’ [40] graded in vivo exposure treatment for youth with chronic pain (called GET Living) may be one such intervention that encompasses these treatment strategies.

Another alternative explanation as to how SPT may contribute to the pediatric chronic pain and anxiety relationship can be derived from Holley and colleagues’ [41] conceptual framework of PTSS and chronic pain in children. According to this model, several individual PTSS and chronic pain symptoms (e.g., hyperarousal, avoidance, and attentional biases), as well as shared psychological factors (e.g., anxiety sensitivity, catastrophizing), are probable contributors to the PTSS–chronic pain comorbidity in youth. These symptoms and factors are posited to influence and exacerbate each other; thus contributing to the mutual maintenance of both conditions. For example, children’s catastrophic thoughts about pain may lead to increased avoidance of stimuli, which in turn serves to maintain chronic pain and PTSD symptoms. Previous research has demonstrated that SPT is a general, higher-order factor underlying the pain-related anxiety constructs of anxiety sensitivity, pain anxiety, and pain catastrophizing [16,17]. Thus, SPT may be a psychological construct that underlies several of the individual, anxiety-related psychological factors proposed in Holley and colleagues’ [41] conceptual mutual maintenance model (e.g., catastrophizing and anxiety sensitivity). SPT may interact with several other of the proposed chronic pain symptoms and psychological factors; thus contributing to the co-occurrence of pediatric chronic pain and PTSS. However, SPT has not been extensively studied as a potential anxiety-related psychological construct in children because a psychological tool to measure the construct in children with chronic pain has only recently been developed [18].

Future prospective research should investigate whether SPT is predictive of pain outcomes in children who are at risk of developing chronic pain across the period of time in which pain transitions from an acute to a chronic state (e.g., in children scheduled for major surgery or children who are at risk for developing chronic post-surgical pain). Replication of Kleiman and colleagues’ [16] study demonstrating that SPT score pre-surgery distinguished between individuals who developed chronic pain versus those who did not develop chronic pain one year post-surgery is also needed in youth samples. Finally, studies testing Holley and colleagues’ [41] model with SPT included are necessary to determine whether SPT is a psychological construct that contributes to the PTSS–chronic pain relationship in youth.

The current study contains several limitations. Firstly, the sample was fairly homogenous, in that the majority of participants were white and girls. This may limit the generalizability of the study’s results to all youth with chronic pain. Participants were also recruited from tertiary chronic pain clinics, so results may not generalize to youth who are not seeking or receiving tertiary treatment for their chronic pain. Secondly, there was some attrition of participants and increased incomplete questionnaire data from baseline to time 2, which resulted in the exclusion of 40 participants from the prospective regression analyses (an approximately 22% loss of sample size). This may have reduced the power of the regression analyses to detect a significant moderation effect. Additional participants would allow us to more confidently assess whether the non-significant results in our study are reflective of true null effects or were due to study limitations. For instance, the non-significant moderating effect of SPT on the relationship between baseline anxiety and pain interference at time 2 may be attributable to pain interference measurement limitations on the Youth Daily Survey, rather than a true null effect. Given that two of the four pain interference items in the survey inquired about mobility-related pain interference (i.e., participants’ ability to run and walk one block), and that most participants (72%) reported headache as their chronic pain condition, it is possible that most participants’ mobility is relatively unaffected by their chronic pain and, in turn, that they reported low pain interference on the Survey. Differing levels of SPT are unlikely to moderate the relationship between anxiety and pain interference when pain interference is low for most participants. Similarly, the significantly lower level of pain unpleasantness at time 2 for participants who completed the study compared to attrition participants may have biased the model towards a non-significant effect.

Lastly, the current study solely relied on self-report data, which may have resulted in biases or errors in responding to questionnaire items (e.g., participants misinterpreting a question, response bias, and so forth). However, because all pain and anxiety scores were determined by averaging participants’ daily ratings over 7 consecutive days, it is reasonable to conclude that at least recall bias was effectively reduced in the current study. Future pediatric questionnaire-based pain studies may reduce potential question misinterpretation by tailoring survey design to the cognitive and social development of differently aged respondents (e.g., through the addition of visual aids and audio presentation of questions for younger respondents), and by ensuring that questions and instructions are clear, simple, unambiguous, and avoid negations [42]. In addition, given that the proximity of siblings and parents can influence participants’ responses, demand characteristics may be reduced if researchers ensure that respondents have privacy when answering questionnaires [42].

## 5. Conclusions

In conclusion, in this clinical sample of youth with chronic pain, SPT moderated the longitudinal relationship between anxiety and pain intensity. Youth who had high anxiety and were more sensitive to experiencing their pain as traumatizing were more likely to experience high pain intensity three months later than youth who had high anxiety but were less sensitive to experiencing their pain as traumatizing. Replication of the current study’s results in larger samples taken from more diverse populations will be necessary to confirm the moderating effect of SPT on the anxiety–pain relationship in youth with chronic pain. Nonetheless, current results suggest that assessing children’s propensity to become traumatized by their pain may help predict pain trajectories. Therefore, SPT may be an important target of intervention when treating chronic pain in youth and may be important to assess in youth at risk of developing chronic pain, such as children preparing for major surgery.

## Figures and Tables

**Table 1:** Sociodemographic and pain characteristics of the sample.

Characteristic	Percentage	Number
Age, *M* = 14.21 years, *SD =* 2.23, range = 10–18		
Gender		
Girl	71	130
Boy	25	46
Nondisclosed	4	8
Race/Ethnicity		
White	79	145
Bi- or multiracial	8	14
Other or declined to answer	4	8
Arab/West Asian	2	4
Black	2	3
South Asian	2	3
Latin American	1	2
Indigenous	1	2
Filipino	<1	1
Pain duration, *M* = 2.69 years, *SD* = 3.04, range = 0.25–12		
Pain locations *		
Head	72	126
Other	25	43
Muscle and joints	24	42
Stomach	18	32
Legs	14	25
Chest	11	19

Note. * Percentages add up to greater than 100 as 40% of youth endorsed multiple pain locations.

**Table 2:** Average daily pain and anxiety characteristics of the sample.

Characteristic	Baseline (Time 1)		Time 2	
	Mean (SD)	Sample Range	Mean (SD)	Sample Range
Anxiety	3.22 (2.11)	0–9	-	-
Pain Intensity	4.55 (2.29)	0–8.71	3.92 (2.52)	0–9.43
Pain Unpleasantness	1.61 (0.77)	0–3.57	1.36 (0.84)	0–3.57
Pain Interference	5.09 (4.02)	0–15.83	4.35 (4.13)	0–16

**Table 3:** Zero-order correlations among variables.

	Baseline Anxiety	Baseline Pain Intensity	Baseline Pain Unpleasantness	Baseline Pain Interference	Baseline SPT	Time 2 Pain Intensity	Time 2 Pain Unpleasantness	Time 2 Pain Interference
Baseline anxiety	1							
Baseline pain intensity	0.39	1						
Baseline pain unpleasantness	0.3	0.73	1					
Baseline pain interference	0.40	0.58	0.68	1				
Baseline SPT	0.37	0.28	0.37	0.47	1			
Time 2 pain intensity	0.34	0.70	0.57	0.49	0.28	1		
Time 2 pain unpleasantness	0.42	0.45	0.6	0.55	0.39	0.74	1	
Time 2 pain interference	0.32	0.35	0.43	0.73	0.36	0.55	0.62	1

Note: All correlations significant at the *p* < 0.01 level.

**Table 4:** Summary of multiple linear regression analyses for variables predicting pain measures.

	Pain Measure	
Predictor	*Adj. R* ^2^	*b*
Model 1	0.48 ***	
Pain intensity		0.69 ***
Baseline anxiety		–0.15
SPT		–0.059
Baseline anxiety x SPT		0.02 *
*n*		
Model 2	0.41 ***	
Pain unpleasantness		0.10 ***
Baseline anxiety		0.005
SPT		–0.002
Baseline anxiety x SPT		0.001
*n*		
Model 3	0.47 ***	
Pain interference		0.058 ***
Baseline anxiety		–0.058
SPT		–0.018
Baseline anxiety x SPT		0.025
*n*		

* p < 0.05. ** *p* < 0.01. *** *p* < 0.001.

## Data Availability

The data presented in this study are available on request from the corresponding author. The data are not publicly available due to privacy considerations.

## Supplementary Materials

The following supporting information can be downloaded at: https://www.mdpi.com/article/10.3390/children9040529/s1, Results S1: Multilevel model methods and results [43].

## References

[1] King S., Chambers C.T., Huguet A., MacNevin R.C., McGrath P.J., Parker L., MacDonald A.J. (2011). The epidemiology of chronic pain in children and adolescents revisited: A systematic review. Pain.

[2] Sato A.F., Hainsworth K.R., Khan K.A., Ladwig R.J., Weisman S.J., Davies W.H. (2007). School absenteeism in pediatric chronic pain: Identifying lessons learned from the general school absenteeism literature. Child. Healthc..

[3] Meldrum M.L., Tsao J.C., Zeltzer L.K. (2008). “Just be in pain and just move on”: Functioning limitations and strategies in the lives of children with chronic pain. J. Pain Manag..

[4] Huguet A., Miró J. (2008). The severity of chronic pediatric pain: An epidemiological study. J. Pain.

[5] Fearon P., Hotopf M. (2001). Relation between headache in childhood and physical and psychiatric symptoms in adulthood: National birth cohort study. BMJ.

[6] Vinall J., Pavlova M., Asmundson G.J., Rasic N., Noel M. (2016). Mental health comorbidities in pediatric chronic pain: A narrative review of epidemiology, models, neurobiological mechanisms and treatment. Children.

[7] Cornwall A., Donderi D. (1988). The effect of experimentally induced anxiety on the experience of pressure pain. Pain.

[8] Benore E., D’Auria A., Banez G.A., Worley S., Tang A. (2015). The influence of anxiety reduction on clinical response to pediatric chronic pain rehabilitation. Clin. J. Pain.

[9] Krishnan K.R.R., France R.D., Pelton S., McCann U.D., Davidson J., Urban B.J. (1985). Chronic pain and depression. II. Symptoms of anxiety in chronic low back pain patients and their relationship to subtypes of depression. Pain.

[10] Asmundson G.J., Katz J. (2009). Understanding the co-occurrence of anxiety disorders and chronic pain: State-of-the-art. Depress. Anxiety.

[11] Jordan K.D., Okifuji A. (2011). Anxiety disorders: Differential diagnosis and their relationship to chronic pain. J. Pain Palliat. Care Pharmacother..

[12] Knook L.M.E., Konijnenberg A.Y., van der Hoeven J., Kimpen J.L.L., Buitelaar J.K., van Engeland H., de Graeff-Meeder E.R. (2011). Psychiatric disorders in children and adolescents presenting with unexplained chronic pain: What is the prevalence and clinical relevancy?. Eur. Child Adolesc. Psychiatry.

[13] Kashikar-Zuck S., Parkins I.S., Graham T.B., Lynch A.M., Passo M., Johnston M., Schikler K.N., Hashkes P.J., Banez G., Richards M.M. (2008). Anxiety, mood, and behavioral disorders among pediatric patients with juvenile fibromyalgia syndrome. Clin. J. Pain.

[14] Campo J.V., Bridge J., Ehmann M., Altman S., Lucas A., Birmaher B., Lorenzo C.D., Iyengar S., Brent D.A. (2004). Recurrent abdominal pain, anxiety, and depression in primary care. Pediatrics.

[15] Rogers A.H., Bakhshaie J., Ditre J.W., Manning K., Mayorga N.A., Viana A.G., Zvolensky M.J. (2019). Worry and rumination: Explanatory roles in the relation between pain and anxiety and depressive symptoms among college students with pain. J. Am. Coll. Health.

[16] Kleiman V., Clarke H., Katz J. (2011). Sensitivity to pain traumatization: A higher-order factor underlying pain-related anxiety, pain catastrophizing and anxiety sensitivity among patients scheduled for major surgery. Pain Res. Manag..

[17] Katz J., Fashler S.R., Wicks C., Pagé M.G., Roosen K.M., Kleiman V., Clarke H. (2017). Sensitivity to Pain Traumatization Scale: Development, validation, and preliminary findings. J. Pain Res..

[18] Pavlova M., Beveridge J., Soltani S., Maunder L., Salomons T., Katz J., Noel M. (2022). The sensitivity to pain traumatization scale-child version (SPTS-C): Development and preliminary validation. Article Submitted for Publication.

[19] Engelhard I.M., van den Hout M.A., Kindt M. (2003). The relationship between neuroticism, pre-traumatic stress, and post-traumatic stress: A prospective study. Personal. Individ. Differ..

[20] Guo W., Xue J.-M., Shao D., Long Z.-T., Cao F.-L. (2015). Effect of the interplay between trauma severity and trait neuroticism on posttraumatic stress disorder symptoms among adolescents exposed to a pipeline explosion. PLoS ONE.

[21] Pagé M.G., Stinson J., Campbell F., Isaac L., Katz J. (2013). Identification of pain-related psychological risk factors for the development and maintenance of pediatric chronic postsurgical pain. J. Pain Res..

[22] Beveridge J.K., Pavlova M., Katz J., Noel M. (2021). The Parent Version of the Sensitivity to Pain Traumatization Scale (SPTS-P): A preliminary validation. Children.

[23] Nelson S., Beveridge J.K., Mychasiuk R., Noel M. (2021). Adverse childhood experiences (ACEs) and internalizing mental health, pain, and quality of life in youth with chronic pain: A longitudinal examination. J. Pain.

[24] Neville A., Griep Y., Palermo T.M., Vervoort T., Schulte F., Yeates K.O., Sumpton J.E., Mychasiuk R., Noel M. (2020). A “dyadic dance”: Pain catastrophizing moderates the daily relationships between parent mood and protective responses and child chronic pain. Pain.

[25] Neville A., Kopala-Sibley D.C., Soltani S., Asmundson G.J., Jordan A., Carleton R.N., Yeates K.O., Schulte F., Noel M. (2021). A longitudinal examination of the interpersonal fear avoidance model of pain: The role of intolerance of uncertainty. Pain.

[26] Pavlova M., Kopala-Sibley D.C., Nania C., Mychasiuk R., Christensen J., McPeak A., Tomfohr-Madsen L., Katz J., Palermo T.M., Noel M. (2020). Sleep disturbance underlies the co-occurrence of trauma and pediatric chronic pain: A longitudinal examination. Pain.

[27] Harris P.A., Taylor R., Thielke R., Payne J., Gonzalez N., Conde J.G. (2009). Research electronic data capture (REDCap)—A metadata-driven methodology and workflow process for providing translational research informatics support. J. Biomed. Inform..

[28] Crandall M., Lammers C., Senders C., Savedra M., Braun J.V. (2007). Initial validation of a numeric zero to ten scale to measure children's state anxiety. Anesth. Analg..

[29] Castarlenas E., Jensen M.P., von Baeyer C.L., Miró J. (2017). Psychometric properties of the numerical rating scale to assess self-reported pain intensity in children and adolescents. Clin. J. Pain.

[30] Pagé M.G., Katz J., Stinson J., Isaac L., Martin-Pichora A.L., Campbell F. (2012). Validation of the numerical rating scale for pain intensity and unpleasantness in pediatric acute postoperative pain: Sensitivity to change over time. J. Pain.

[31] Palermo T.M., Valenzuela D., Stork P.P. (2004). A randomized trial of electronic versus paper pain diaries in children: Impact on compliance, accuracy, and acceptability. Pain.

[32] Noel M., Rabbitts J.A., Tai G.G., Palermo T.M. (2015). Remembering pain after surgery: A longitudinal examination of the role of pain catastrophizing in children’s and parents’ recall. Pain.

[33] Varni J.W., Stucky B.D., Thissen D., DeWitt E.M., Irwin D., Lai J.-S., Yeatts K., DeWalt D.A. (2010). PROMIS Pediatric Pain Interference Scale: An item response theory analysis of the Pediatric Pain Item Bank. J. Pain.

[34] Kashikar-Zuck S., Carle A., Barnett K., Goldschneider K.R., Sherry D.D., Mara C.A., Cunningham N., Farrell J., Tress J., DeWitt E.M. (2016). Longitudinal evaluation of Patient Reported Outcomes Measurement Information Systems (PROMIS) measures in pediatric chronic pain. Pain.

[35] Aiken L., West S. (1991). Multiple Regression: Testing and Interpreting Interactions.

[36] Johnson P.O., Fay L.C. (1950). The Johnson-Neyman technique, its theory and application. Psychometrika.

[37] Scholz J., Woolf C.J. (2002). Can we conquer pain?. Nat. Neurosci..

[38] Foa E.B., Kozak M.J. (1986). Emotional processing of fear: Exposure to corrective information. Psychol. Bull..

[39] Bosco M.A., Gallinati J.L., Clark M.E. (2013). Conceptualizing and treating comorbid chronic pain and PTSD. Pain Res. Treat..

[40] Simons L.E., Vlaeyen J.W., Declercq L., Smith A.M., Beebe J., Hogan M., Li E., Kronman C.A., Mahmud F., Corey J.R. (2020). Avoid or engage? Outcomes of graded exposure in youth with chronic pain using a sequential replicated single-case randomized design. Pain.

[41] Holley A., Wilson A., Noel M., Palermo T. (2016). Post-traumatic stress symptoms in children and adolescents with chronic pain: A topical review of the literature and a proposed framework for future research. Eur. J. Pain.

[42] Borgers N., de Leeuw E., Hox J. (2000). Children as respondents in survey research: Cognitive development and response quality 1. Bull. Sociol. Methodol./Bull. Méthodologie Sociol..

[3] Pinheiro J., Bates D., DebRoy S., Sarkar D., R Core Team (2021). Nlme: Linear and Nonlinear Mixed Effects Models. R Package Version 3.1-153. https://CRAN.R-project.org/package=nlme.

