# Supplementary material for: Sensitivity to Pain Traumatization and Its Relationship to the Anxiety–Pain Connection in Youth with Chronic Pain: Implications for Treatment
Source: Children (Basel). 2022 Apr 8;9(4):529. doi: 10.3390/children9040529 (PMC9032504; doi:10.3390/children9040529)
Supplement: Supplementary file 1 [file children-09-00529-s001.zip › children-1631769-supplementary.pdf]

Supplemental Data: Multilevel Random Coefficient Models

Methods

Statistical Analyses

Given our interest in temporal relations between SPT and the anxiety-pain relationship, we investigated whether SPT moderated the pain-anxiety relationship on a day-to-day basis. To do so, three multilevel models were employed to determine whether daily anxiety ratings were significantly predictive of next-day pain ratings (i.e., daily pain intensity, pain unpleasantness, and pain interference), at baseline and at time 2. RStudio Version 1.1.383 was used to build all multilevel models. For all three models, daily anxiety ratings were employed as level 1 predictor. Daily pain intensity, unpleasantness, and interference were employed as criterion variable (in three separate models). To reduce the possibility of reciprocal causation, daily anxiety observations were regressed with pain ratings recorded the next day (e.g., anxiety ratings on day 1 were regressed with pain ratings on day 2). SPT score was used as level 2 predictor in the multilevel models to determine whether participants' propensity to become traumatized by their pain significantly predicated variation in mean pain rating (i.e., intercept-variation). To determine whether SPT significantly predicted variation in the anxiety-pain relationship (i.e., anxiety-pain slope), one two-way cross-level interaction was included in each of the three models: daily anxiety x STP score. Each model was conducted twice, once for survey data from baseline, and again for survey data from time 2. Thus, six 2-level multilevel random coefficient models were conducted, using the nlme package in R [1]. Because the residuals of the criterion variables of pain intensity, unpleasantness, and interference were non-normally distributed for both baseline and time 2 models, baseline and time 2 pain intensity, unpleasantness, and interference were log transformed. For all multilevel models, listwise deletion of cases was applied to missing values.

Results

Baseline Models Build-up

Model 1: Pain Intensity

This study acquired multiple pain ratings from the same participant, thus it was likely that responses from each participant would tend to be related. Therefore, to determine how much variance in daily pain intensity was attributable to group membership (each participant representing one group), a null model with daily pain intensity as criterion variable and with no predictors was conducted. This allowed for calculation of the intraclass correlation coefficient (ICC). For pain intensity, the ICC was .64, suggesting that 64% of the variance in daily pain intensity was explained by the effect of group membership on pain intensity (i.e., by scores being related by virtue of coming from the same participant). Because the ICC for pain intensity was greater than .10, there was a significant amount of between-group variance, and a multilevel model was thus appropriate to use for the model data.

Next, a random-intercepts model with the level 1 variable of daily anxiety predicting daily pain intensity was employed (Level 1 Random-Intercepts Model), to determine whether there was a significant relationship between daily anxiety and next-day pain intensity. Results demonstrated that there was a significant relationship between daily anxiety and pain intensity the next day, b = .010, $t(820) = 2.14$, $p = .032$. The Level 1 Random-Intercepts Model was updated by allowing the daily anxiety-daily pain intensity slopes to randomly vary between participants. An ANOVA was then carried out between the Level 1 Random-Intercepts Model and Level 1 Random-Slopes + Random-Intercepts Model to determine whether the latter model accounted for a significantly greater amount of variance than the model which did not allow slopes to randomly vary. The analysis of variance indicated that the Level 1 Random-Intercepts Model fit the data significantly

better than the Level 1 Random-Slopes + Random-Intercepts model. Therefore, we employed a random-intercepts, fixed-slopes model (i.e., henceforth the Level 1 Model) in subsequent analyses on pain intensity.

Error structures were next assessed to determine whether a model that allowed for autocorrelation of data improved model fit. Autocorrelation was modeled by adding the corAR1() function to the Level 1 Model. The autocorrelation estimate (Phi coefficient) was .10, and an ANOVA conducted between the Level 1 Model and Level 1 Model that allowed for autocorrelation showed that the model which allowed for autocorrelation fit the data significantly better than the Level 1 Model alone, $p = .026$. A preliminary examination of the variance of pain intensity on each day did not suggest that the variance of pain intensity observations increased or decreased over time; therefore, homogeneity of variance was assumed, and the model was not updated to allow for heterogeneity in error structures.

Finally, the level 2 predictor of SPT and two-way cross-level interaction of interest (daily anxiety x SPT) were added to the Level 1 Model with corAR1(). In this final model, daily anxiety was not significantly related to daily pain intensity, $b = .0060$, $t(782) = 1.09$, $p = .28$. The level 2 predictor, SPT, was significantly related to mean pain intensity (i.e., pain intensity averaged over the 7-day study period), $b = .0064$, $t(171) = 2.67$, $p = .008$. Participants' mean daily anxiety was significantly different from zero, $p < .001$. When the model was conducted without log correction of pain intensity (for interpretability purposes), participants' mean daily anxiety was 3.58/10. SPT did not significantly explain variation in the daily anxiety – daily pain intensity slope. In other words, the 2-way interaction between daily anxiety and SPT was not significant, $p > .2$.

Model 2: Pain Unpleasantness

The null model with daily pain unpleasantness employed as criterion variable and with no predictors produced an ICC of .46. This suggested that 46% of the variance in daily pain unpleasantness was attributable to group membership, and that a multilevel model was appropriate to use for the model data. The Level 1 Random-Intercepts Model with daily anxiety predicting daily pain unpleasantness demonstrated that there was no significant relationship between daily anxiety and next-day pain unpleasantness, $p > .20$. After the Level 1 Random-Intercepts Model was updated to allow slopes to vary, an ANOVA comparing the Level 1 Random-Intercepts Model and Level 1 Random-Slopes + Random-Intercepts Model indicated that the Level 1 Random-Intercepts Model fit the data significantly better than the Level 1 Random-Slopes + Random-Intercepts model. Therefore, the random-intercepts, fixed-slopes model was used in subsequent analyses on pain unpleasantness.

Autocorrelation was modeled, which produced an autocorrelation estimate of .095. An ANOVA conducted between the Level 1 Model and Level 1 Model that allowed for autocorrelation demonstrated that there was significant autocorrelation in the data, $p = .046$. A preliminary examination of the variance of pain unpleasantness on each day suggested no systematic increases or decreases in the variance of pain unpleasantness observations over time; therefore, homogeneity of variance was assumed.

The level 2 predictor of SPT and two-way cross-level interaction of interest (daily anxiety x SPT) were added to the Level 1 Model with corAR1(). In this final model, daily anxiety was not significantly related to daily pain unpleasantness, $p > .20$. The level 2 predictor, SPT, was significantly related to mean pain unpleasantness, $b = .0057$, $t(169) = 3.95$, $p < .001$. Participants' mean daily anxiety was significantly different from zero, $p < .001$. When the model was conducted without log correction of pain unpleasantness (for interpretability purposes), participants' mean

daily anxiety was 1.20/10. SPT did not significantly explain variation in the daily anxiety – daily pain unpleasantness slope, *p* > .20.

Model 3: Pain Interference

The null model produced an ICC of .74, indicating that 74% of the variance in daily pain interference was explained by the effect of group membership. The Level 1 Random-Intercepts Model with daily anxiety predicting daily pain interference showed a significant relationship between daily anxiety and pain interference the next day, *b* = .012, *t*(730) = 2.77, *p* = .006. After the Level 1 Random-Intercepts Model was updated to allow slopes to vary, an ANOVA comparing the Level 1 Random-Intercepts Model and Level 1 Random-Slopes + Random-Intercepts Model indicated that the Level 1 Random-Intercepts Model fit the data significantly better than the Level 1 Random-Slopes + Random-Intercepts model. Therefore, the random-intercepts, fixed-slopes model was used in subsequent analyses on pain interference.

Autocorrelation was modeled, which produced an autocorrelation estimate of .31. An ANOVA conducted between the Level 1 Model and Level 1 Model that allowed for autocorrelation demonstrated that there was significant autocorrelation in the data, *p* < .001. A preliminary examination of the variance of pain interference suggested no systematic increases or decreases in the variance of pain interference observations over time; therefore, homogeneity of variance was assumed.

The level 2 predictor of SPT and two-way cross-level interaction of interest (daily anxiety x SPT) were added to the Level 1 Model with corAR1(). In this final model, daily anxiety was not significantly related to daily pain interference, *p* > .20. The level 2 predictor, SPT, was significantly related to mean pain interference, *b* = .015, *t*(168) = 4.73, *p* < .001. Participants' mean daily anxiety was significantly different from zero, *p* < .001. When the model was conducted

without log correction of pain interference (for interpretability purposes), participants' mean daily anxiety was 2.23/10. SPT did not significantly explain variation in the daily anxiety – daily pain interference slope, $p > .20$.

Time 2 Models Build-up

Model 1: Pain Intensity

The null model produced an ICC of .70, indicating that 70% of the variance in daily pain intensity at time 2 was explained by the effect of group membership. Like at baseline, the Level 1 Random-Intercepts Model with daily anxiety predicting daily pain intensity showed a significant relationship between daily anxiety and next-day pain intensity, $b = .0075$, $t(685) = 1.98$, $p = .048$. The ANOVA comparing the Level 1 Random-Intercepts Model and Level 1 Random-Slopes + Random-Intercepts Model indicated that the Level 1 Random-Intercepts Model fit the data significantly better, so the Random-Intercepts Model was retained in subsequent analyses. The autocorrelation estimate was .18. An ANOVA conducted between the Level 1 Model and Level 1 Model that allowed for autocorrelation demonstrated that there was significant autocorrelation in the data, $p < .001$. Examination of the variance of pain intensity suggested that homogeneity of variance could be assumed. The level 2 predictor of SPT and the two-way cross-level daily anxiety x SPT interaction were added to the Level 1 Model with corAR1(). In this final model, daily anxiety was not significantly related to daily pain intensity, $p > .20$. The level 2 predictor, SPT, was not significantly related to mean pain intensity, $b = .004$, $t(146) = 1.50$, $p = .13$. Participants' mean daily anxiety was significantly different from zero, $p < .001$. When the model was conducted without log correction of pain intensity, participants' mean daily anxiety was 3.12/10. The 2-way interaction between daily anxiety and SPT was not significant, $b = 0.0005$, $t(664) = 1.37$, $p = .17$.

Model 2: Pain Unpleasantness

The null model produced an ICC of .52, indicating that 52% of the variance in daily pain unpleasantness at time 2 was attributable to group membership. The Level 1 Random-Intercepts Model with daily anxiety predicting daily pain unpleasantness showed a significant relationship between daily anxiety and pain unpleasantness the next day, b = .0061, $t(578)$ = 2.02, p = .043. The ANOVA comparing the Level 1 Random-Intercepts Model and Level 1 Random-Slopes + Random-Intercepts Model indicated that the Level 1 Random-Intercepts Model fit the data significantly better, so the Random-Intercepts Model was retained in subsequent analyses. The autocorrelation estimate was .11. An ANOVA conducted between the Level 1 Model and Level 1 Model that allowed for autocorrelation demonstrated that the model allowing for autocorrelation did not fit the data significantly better, p = .06, so the Level 1 Model without autocorrelation was retained in subsequent analyses. Examination of the variance of pain unpleasantness suggested that homogeneity of variance could be assumed. The level 2 predictor of SPT and the two-way cross-level daily anxiety x SPT interaction were added to the Level 1 Model. In this final model, daily anxiety was not significantly related to daily pain unpleasantness, $p > .20$. The level 2 predictor, SPT, was significantly related to mean pain unpleasantness, b = .004, $t(141)$ = 2.47, p = .01. Participants' mean daily anxiety was significantly different from zero, $p < .001$. When the model was conducted without log correction of pain unpleasantness, participants' mean daily anxiety was 0.94/10. The 2-way interaction between daily anxiety and SPT was not significant, b = 0.0005, $t(556)$ = 1.69, p = .09.

Model 3: Pain Interference

The null model produced an ICC of .84, indicating that 84% of the variance in daily pain interference at time 2 was attributable to group membership. The Level 1 Random-Intercepts Model with daily anxiety predicting next-day pain interference was not significant, b = .006, $t(571)$

= 1.44, p = .15. The ANOVA comparing the Level 1 Random-Intercepts Model and Level 1 Random-Slopes + Random-Intercepts Model indicated that the Level 1 Random-Intercepts Model fit the data significantly better, so the Random-Intercepts Model was retained in subsequent analyses. The autocorrelation estimate was .13. An ANOVA conducted between the Level 1 Model and Level 1 Model that allowed for autocorrelation demonstrated that the model which allowed for autocorrelation fit the model data significantly better, $p = .029$. Examination of the variance of pain interference suggested that homogeneity of variance could be assumed. The level 2 predictor of SPT and the two-way cross-level daily anxiety x SPT interaction were added to the Level 1 Model with corAR1(). In this final model, daily anxiety was not significantly related to daily pain interference, $p > .20$. However, SPT was significantly related to mean pain interference, $b = .011$, $t(139) = 3.07$, $p = .003$. Participants' mean daily anxiety was significantly different from zero, $p <$ .001. When the model was conducted without log correction of pain interference, participants' mean daily anxiety was 2.30/10. The 2-way interaction between daily anxiety and SPT was not significant, $p > .20$.

References

[1] Pinheiro J, Bates D, DebRoy S, Sarkar D, R Core Team (2021). *nlme: Linear and Nonlinear Mixed Effects Models*. R package version 3.1-153, https://CRAN.R-project.org/package=nlme.
